# Supplementary material for: Mushroom body output neurons MBON-a1/a2 define an odor intensity channel that regulates behavioral odor discrimination learning in larval Drosophila
Source: Front Physiol. 2023 May 15;14:1111244. doi: 10.3389/fphys.2023.1111244 (PMC10225628; doi:10.3389/fphys.2023.1111244)
Supplement: Supplementary file 1 [file Presentation1.pdf]

Supplementary Material

Mushroom body output neurons MBON-a1/a2 define an odor intensity channel that regulates behavioral odor discrimination learning in larval *Drosophila*.

Abdulkadir Mohamed † Iro Malekou †, Timothy Sim, Cahir J. O'Kane, Yousef Maait, Benjamin Scullion, and Liria M. Masuda-Nakagawa*

***correspondence:** Corresponding Author: lm546@cam.ac.uk

1 Supplemental Data:

Supplementary Data File 1.
Synapse numbers of neurons innervating MBON-a1/a2 in the calyx. Using the synapse numbers calculated in Supplementary Data File 2, the synapse numbers of calyx-innervating neurons were calculated for MBON-a1-R, MBON-a1-L, MBON-a1-R, MBON-a2-L. Percentage from total input was calculated and represented as in Fig. 4.

Supplementary Data File 2.
Localization of synapses of neurons upstream of MBON-a1/a2. Using the connectivity widget of CATMAID, individual synapses of neurons upstream of MBON-a1/a2 were identified by their position on the 3D image visualised by CATMAID. Total number of synapses by a given upstream neuron in the calyx are annotated in column C, synapses in other regions than the calyx in column D. Some synapses were not annotated in the 3D image, and they are recorded as "missing". Some synapses were found in the ipsilateral or contralateral output of MBON-a1/a2. CA: calyx, L1 (proximal) and L2 (distal) are ipsilateral output regions. The division of L1/L2 was made to facilitate counting. CL1, CL2: equivalent contralateral output regions. PE is the pedunculus.

2 Supplementary Figures and Tables

2.1 Supplementary Figures

Supplementary Figure S1. Regions of interest for imaging calyx responses.
Regions of interest (ROIs) were drawn around the edges of the calyx regions in which jRCaMP1b sensor fluorescence was detected, as described in Materials and Methods. Examples are shown of calyces from a larva expressing the sensor using *MB242A* split-GAL4 labeling MBON-a1/a2 (left) or *MB247-GAL4* labeling KCs (right). Panels are 35 µm square.

Supplementary Figure S2. Pattern of calyx innervation by double reporter lines. A. Pattern of MBON-a1/a2 and KC innervation in the calyx. A line expressing *68B12-GAL4* in a single MBON-a1/a2 neuron and *MB247-LexA* in KCs was crossed to reporter line *UAS-mCD8::RFP; LexAop-mCD8::GFP*. Reporter expression was detected by anti-GFP, anti-DsRed and calyx by anti-Dlg. Glomeruli are shown by dotted lines. **B.** MBON-a1/a2 expressing *52E12-GAL4* and PNs expressing *GH146-LexA*, showing native fluorescence from the same double reporter. **C.**

APL neuron expressing *26G02-LexA* and MBON-a1/a2 expressing *52E12-GAL4,* showing native fluorescence from the double reporter. **D.** Stereo image of *OK263-GAL4* line labeling MBON-a1/a2 with CD4::DsRed and nSyb::GFP, with the MB outline shown by anti-Dlg. Panels A-C are single confocal sections of right brain images, anterior to bottom. Panel D is a frontal view of the right brain. Scale bars are 10 µm.

Supplementary Figure S3. GRASP controls.
The GRASP stock *UAS-CD4::spGFP1-10; LexAop-CD4::spGFP11* was crossed to each of the individual *GAL4* and *LexA* lines used for GRASP analysis in Fig. 2. Panels show confocal projections of 3 successive sections through the calyx, labeled using anti-GFP and anti-Dlg. Panels are 35 µm square.

Supplementary Figure S4. Presynaptic and postsynaptic sites at the ipsilateral and contralateral MB output regions visualized using CATMAID.
Output regions of the four MBON-a1/ MBON-a2 neurons analyzed for synaptic counts. In all panels, small cyan circles are MBON-a1-R and MBON-a2-R postsynaptic sites, and small red circles are presynaptic sites. The larger brown and red circles are unfinished tracing sites. Axes are shown at the top right corner of each panel. Panels show processes of **(A)** MBON-a1-R, **(B)** MBON-a2-R, **(C)** MBON-a1-L and **(D)** MBON-a2-L, all on ipsilateral (i) and contralateral (ii) sides.

Supplementary Figure S5. **Synapses of MBON-a1 onto MBONs and MBINs in the output region.**
EM sections of first-instar larva MBON-a1/a2. Panels show MBON-a1-L presynaptic to a number of MBON neurons labeled as MB2ONs, and MBINs. The top left panel shows an EM section with CATMAID annotations; a connector (orange) is placed on the presynaptic neuron and the cyan arrows indicate postsynaptic partners. The top right panel shows the same EM section without the CATMAID annotations. The bottom panel shows an enlargement of the area in the top right panel. Downstream neurons have multiple names which have been omitted in the figure.

Supplementary Figure S6. Example of a KC synapsing with the different regions of MBON-a1/a2 neurons, visualized by CATMAID.
Synapses between KC100-R and three of the MBON-a1/2 neurons are shown as large green dots, pointed by a white arrow. Only one of the calyx synapses is visible. ML, medial lobe projections of MBON-a1-L. Ped, pedunculus.

Supplementary Figure S7. Effects of ChR2-XXL activation in *Tdc2-LexA*-expressing neurons on integrated odor responses in MBON-a1/a2 neurons.
The data presented in Figure 6B-I were reanalyzed and compared as in the graphs of panels 6B-6I, but instead using odor responses integrated from the timepoint when first detected until the end of each recording, rather than peak responses. The main conclusions are similar to those of Fig. 6, namely that the presence or absence of *Tdc2-LexA* makes no significant difference to the effect of ChR2-XXL activation. In contrast to Fig. 6 there is now a slightly significant effect of ChR2-XXL activation even in the absence of *Tdc2-LexA*, possibly a consequence of including more data in the analysis than just the peak data of Fig. 6.
Normally distributed data are shown with mean ± SEM, otherwise with median and interquartile range. Odor and Light/Odor data were compared using a two-tailed paired t-test when both datasets were normally distributed, otherwise using a Wilcoxon test. Ratio data were

compared using one-way ANOVA with Dunnett's T3 post-hoc test (calyx) or Kruskal-Wallis tests with Dunn's post-hoc test (output region).

Supplementary Figure S8. Light contribution to MBON-a1/a2 activity in brains expressing ChR2-XXL in *Tdc2-LexA*-expressing neurons.
To investigate the contribution of the light response to the odor-evoked response in MBON-a1/a2 in the larvae used for combined optogenetics and imaging in Fig. 6B and Fig. 6F, we compared the response of MBON-a1/a2 to "Light only" and "Odor only" in a set of preparations in which the sequence of (i) Odor only, (ii) Light+Odor was followed by (iii) light only.
A. i. Time courses of MBON-a1/a2 calyx ΔF/F from larvae of the same genotype as Fig 6 B and 6F, in response to odor-only or light-only. **ii.** MBON-a1/a2 responses to odor following activation of ChR2-XXL, and a curve showing the hypothetical ΔF/F values from the sum of odor-only and light-only responses shown in **i**. **iii.** Comparisons of peak ΔF/F responses for odor only, odor followed by light, light only and the calculated sum of Light only and Odor only responses.
B. Genotype, graphs and analyses are as in **A**, but for the MBON-a1/a2 output region.

Supplementary Figure S9. Learning in larvae expressing CsChrimson under control of *MB242A*, using dilute odorant concentrations.
Data were generated and analyzed as for Figure 7, using larval progeny of a cross of *MB242A* split-GAL4 to *UAS-CsChrimson*. No significant effect of amber light, which activates CsChrimson, is found ($P > 0.3$, unpaired t-test).

Supplementary Figure S1

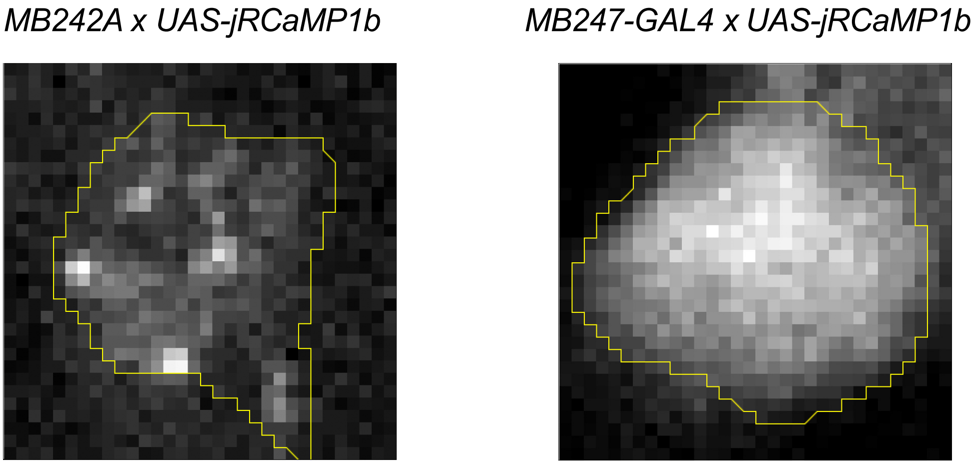

Supplementary Figure S1. Regions of interest for imaging calyx responses. Regions of interest (ROIs) were drawn manually around the edges of the calyx regions in which jRCaMP1b sensor fluorescence was detected. Examples are shown of calyces from a larva expressing the sensor using *MB242A* split-GAL4 labeling MBON-a1/a2 (left) or *MB247-GAL4* labeling KCs (right). Panels are 35 μm square.

Supplementary Figure S2

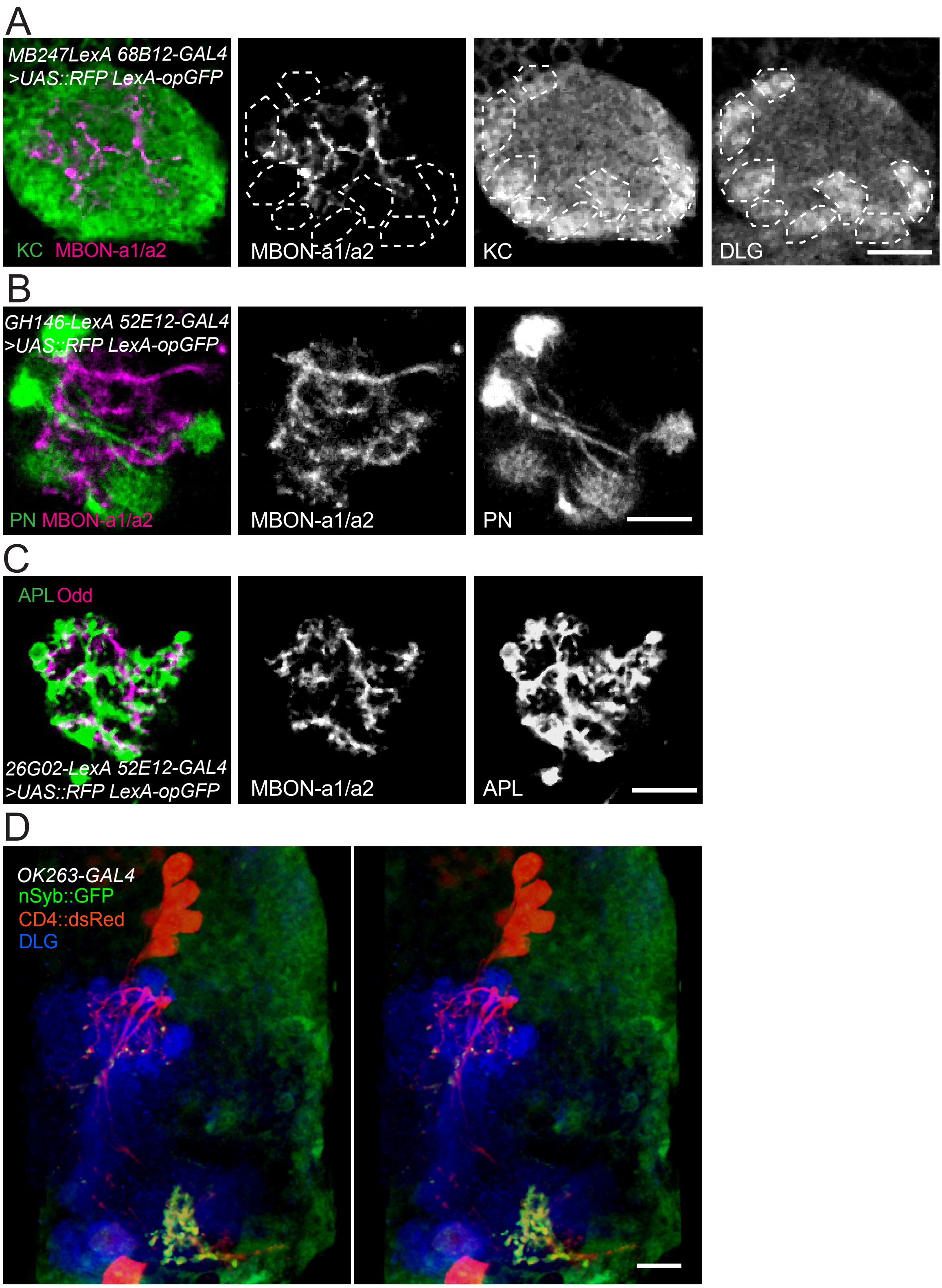

Supplementary Figure S2. Pattern of calyx innervation by double reporter lines. A. Pattern of MBON-a1/a2 and KC innervation in the calyx. A line expressing *68B12-GAL4* in a single MBON-a1/a2 neuron and *MB247-LexA* in KCs was crossed to reporter line *UAS-mCD8::RFP; LexAop-mCD8::GFP*. Reporter expression was detected by anti-GFP, anti-DsRed and calyx by anti-Dlg. Glomeruli are shown by dotted lines. **B.** MBON-a1/a2 expressing *52E12-GAL4* and PNs expressing *GH146-LexA*, showing native fluorescence from the same double reporter. **C.** APL neuron expressing *26G02-LexA* and MBON-a1/a2 expressing *52E12-GAL4,* showing native fluorescence from the double reporter. **D.** Stereo image of *OK263-GAL4* line labeling MBON-a1/a2 with CD4::DsRed and nSyb::GFP, with the MB outline shown by anti-Dlg. Panels A-C are single confocal sections of right brain images, anterior to bottom. Panel D is a frontal view of the right brain. Scale bars are 10 μm.

Supplementary Figure S3

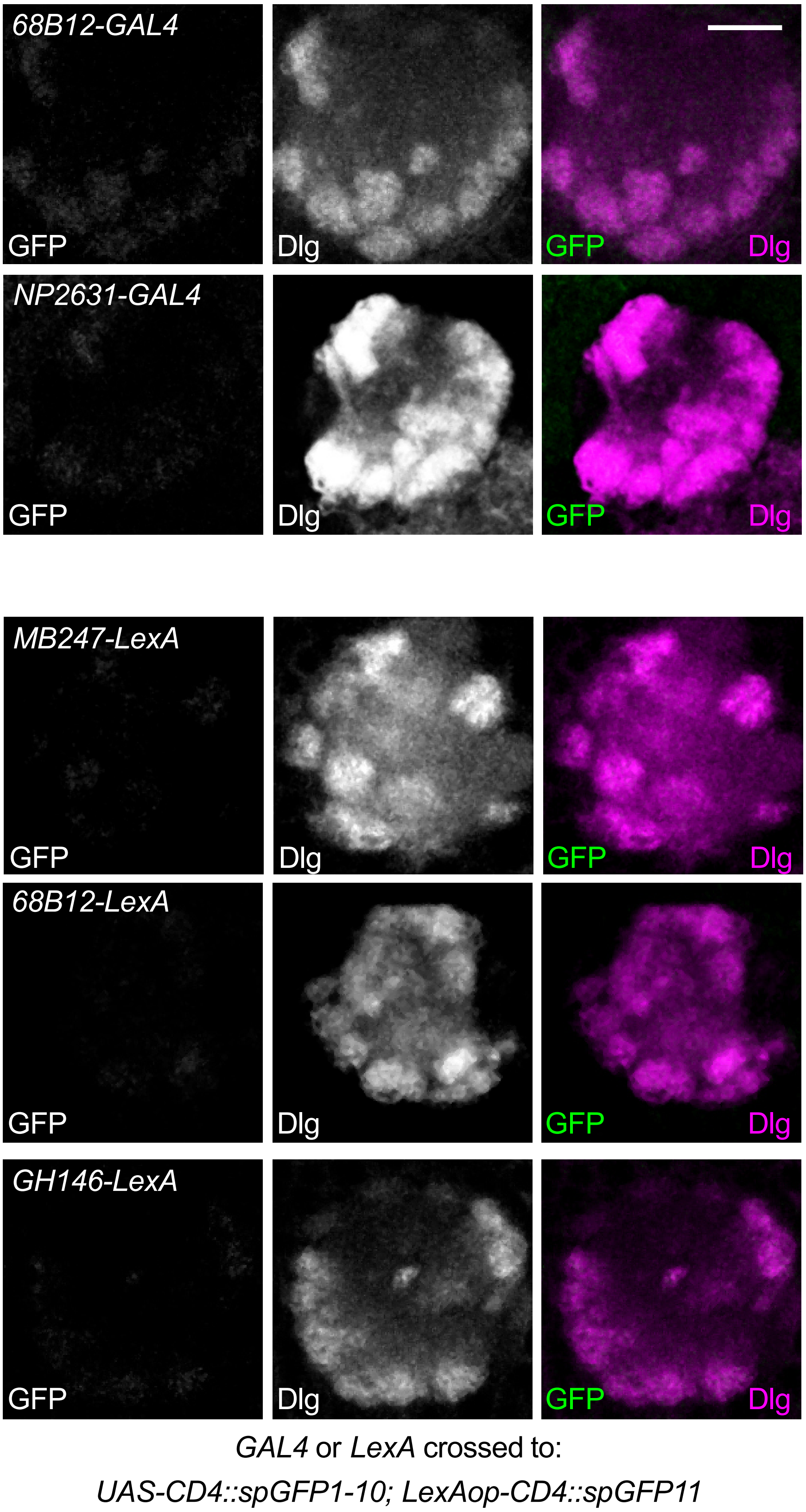

Supplementary Figure S3. GRASP controls. The GRASP stock *UAS-CD4::spGFP1-10; LexAop-CD4::spGFP11* was crossed to each of the individual *GAL4* and *LexA* lines used for GRASP analysis in Fig. 2. Panels show confocal projections of 3 successive sections through the calyx, labeled using anti-GFP and anti-Dlg. Panels are 35 µm square.

Supplementary Figure S4

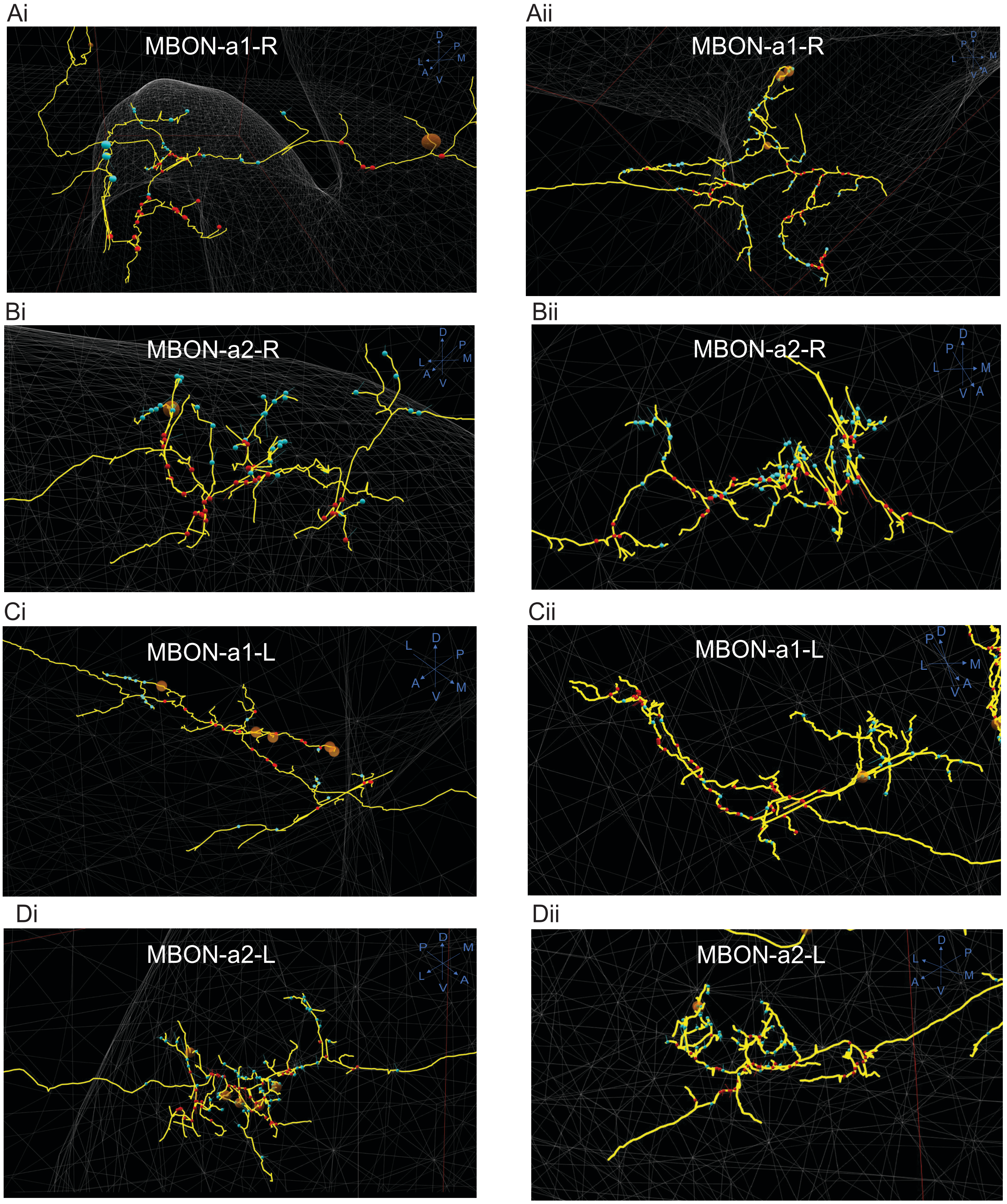

Supplementary Figure S4. Presynaptic and postsynaptic sites at the ipsilateral and contralateral MB output areas visualized using CATMAID.
Output regions of the four MBON-a1/ MBON-a2 neurons analyzed for synaptic counts. In all panels, small cyan circles are MBON-a1-R and MBON-a2-R postsynaptic sites, and small red circles are presynaptic sites. The larger brown and red circles are unfinished tracing sites. Axes are shown at the top right corner of each panel. Panels show processes of **(A)** MBON-a1-R, **(B)** MBON-a2-R, **(C)** MBON-a1-L and **(D)** MBON-a2-L, all on ipsilateral (i) and contralateral (ii) sides.

Supplementary Figure S5

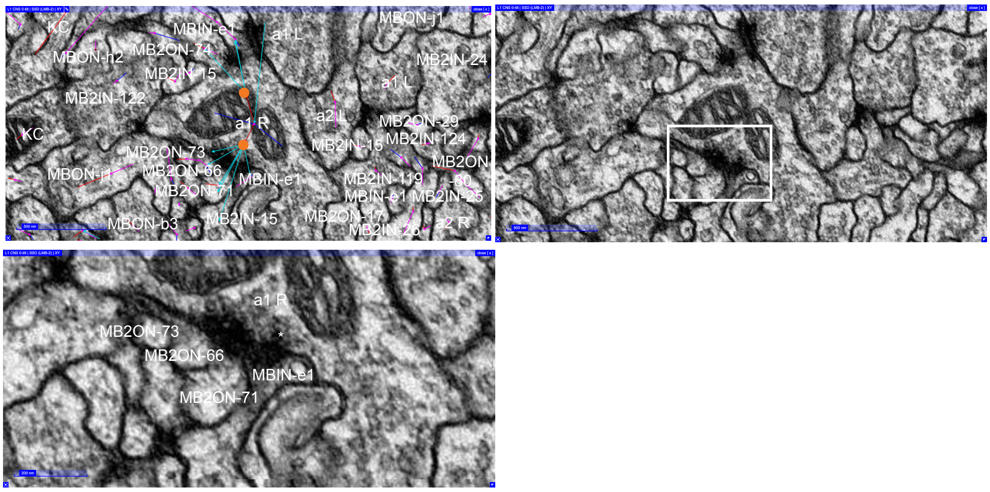

Supplementary Figure S5. **Synapses of MBON-a1 onto MBONs and MBINs in the output region.**

EM sections of first-instar larva MBON-a1/a2. Panels show MBON-a1-L presynaptic to a number of MBON neurons labeled as MB2ONs, and MBINs. The top left panel shows an EM section with CATMAID annotations; a connector (orange) is placed on the presynaptic neuron and the cyan arrows indicate postsynaptic partners. The top right panel shows the same EM section without the CATMAID annotations. The bottom panel shows an enlargement of the area in the top right panel. Downstream neurons have multiple names which have been omitted in the figure.

MBON-a1-R: https://l1em.catmaid.virtualflybrain.org/?pid=1&zp=39750&yp=36333.76014490835&xp=68737.75884963221&tool=tracingtool&sid0=1&s0=-0.8000000000000008

Supplementary Figure S6

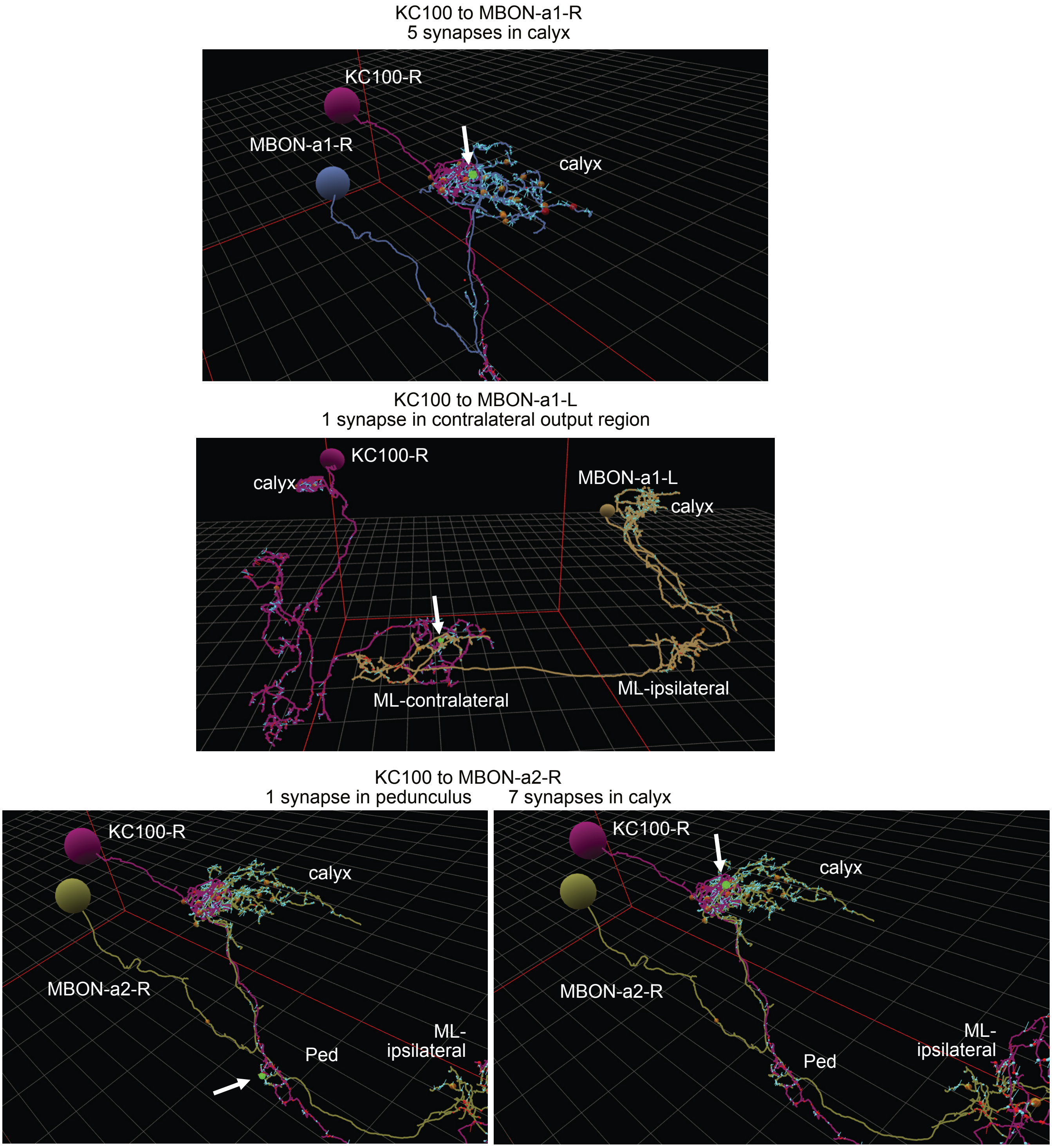

Supplementary Figure S6. Example of a KC synapsing with the different regions of MBON-a1/a2 neurons, visualized by CATMAID.

Synapses between KC100-R and three of the MBON-a1/2 neurons are shown as large green dots, pointed by a white arrow. Only one of the calyx synapses is visible. ML, medial lobe projections of MBON-a1-L. Ped, pedunculus.

Supplementary Figure S7

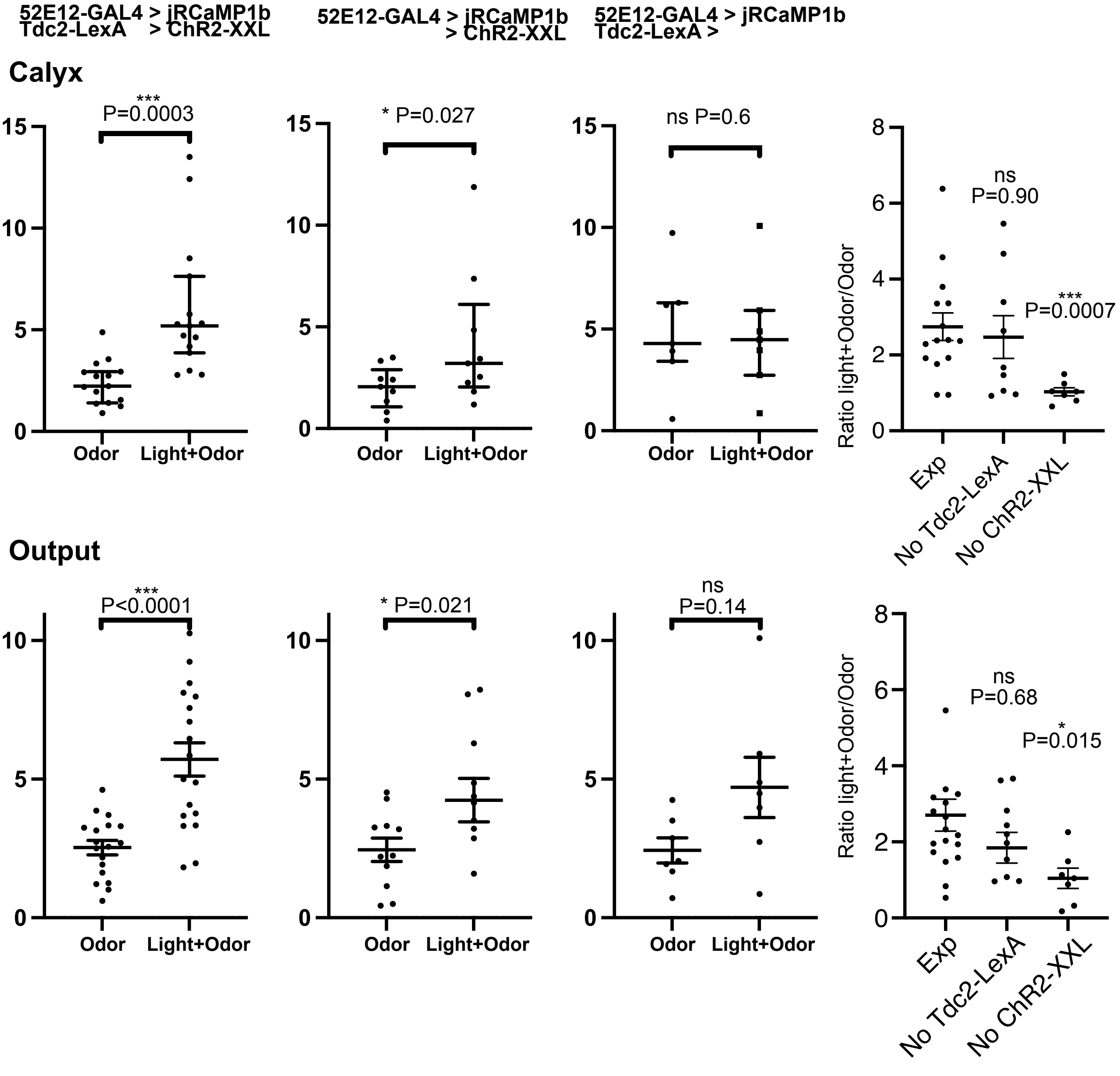

Supplementary Figure S7. Effects of ChR2-XXL activation in *Tdc2-LexA*-expressing neurons on integrated odor responses in MBON-a1/a2 neurons.
The data presented in Figure 6B-I were reanalyzed and compared as in the graphs of panels 6B-6I, but instead using odor responses integrated from the timepoint when first detected until the end of each recording, rather than peak responses. The main conclusions are similar to those of Fig. 6, namely that the presence or absence of Tdc2-LexA makes no significant difference to the effect of ChR2-XXL activation. In contrast to Fig. 6 there is now a slightly significant effect of ChR2-XXL activation even in the absence of Tdc2-LexA, possibly a consequence of including more data in the analysis than just the peak data of Fig. 6.
Normally distributed data are shown with mean ± SEM, otherwise with median and interquartile range. Odor and Light/Odor data were compared using a two-tailed paired t-test when both datasets were normally distributed, otherwise using a Wilcoxon test. Ratio data were compared using one-way ANOVA with Dunnett's T3 post-hoc test (calyx) or Kruskal-Wallis tests with Dunn's post-hoc test (lobe).

Supplementary Figure S8

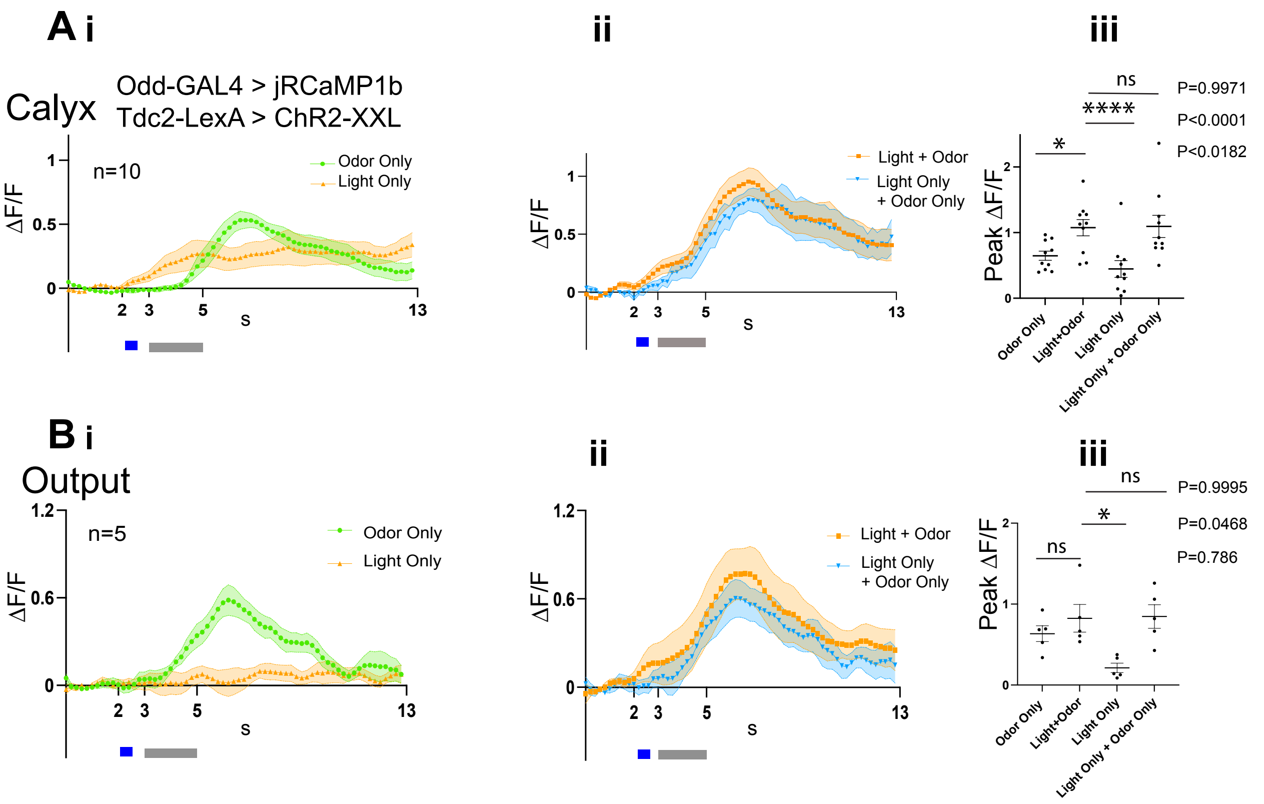

Supplementary Figure S8. Light contribution to MBON-a1/a2 activity in brains expressing ChR2-XXL in *Tdc2-LexA*-expressing neurons.

To investigate the contribution of the light response to the odor-evoked response in MBON-a1/a2 in the larvae used for combined optogenetics and imaging in Fig. 6B and Fig. 6F, we compared the response of MBON-a1/a2 to "Light only" and "Odor only" in a set of preparations in which the sequence of (i) Odor only, (ii) Light+Odor was followed by (iii) light only.

A. i. Time courses of MBON-a1/a2 calyx ΔF/F from larvae of the same genotype as Fig 6 B and 6F, in response to odor-only or light-only. **ii.** MBON-a1/a2 responses to odor following activation of ChR2-XXL, and a curve showing the hypothetical ΔF/F values from the sum of odor-only and light-only responses shown in **i**. **iii.** Comparisons of peak ΔF/F responses for odor only, odor followed by light, light only and the calculated sum of Light only and Odor only responses.

B. Genotype, graphs and analyses are as in **A**, but for the MBON-a1/a2 output region.

Supplementary Figure S9

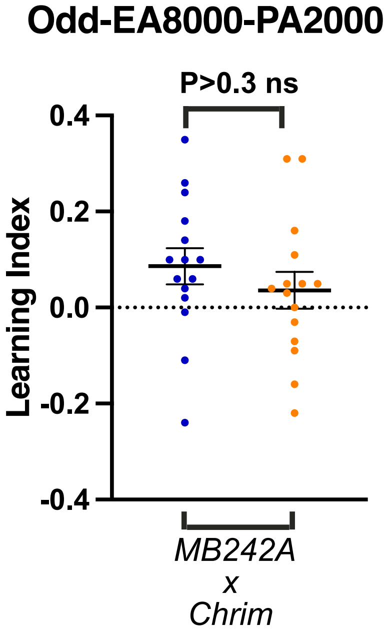

Supplementary Figure S9. Learning in larvae expressing CsChrimson under control of *MB242A*, using dilute odorant concentrations.

Data were generated and analyzed as for Figure 7, using larval progeny of a cross of *MB242A* split-GAL4 to *UAS-CsChrimson*. No significant effect of amber light, which activates CsChrimson, is found (P > 0.3, unpaired t-test).

2.1 Supplementary Tables

Table S1. Presynaptic and postsynaptic sites of MBON-a1/ MBON-a2 in the calyx. The 3D CATMAID reconstructions of MBON-a1-R, MBON-a2-R, MBON-a1-L and MBON-a2-L from a single 6-hour larva were used to make these measurements.

Neurons	Number of presynaptic sites in the calyx	Number of postsynaptic sites in the calyx	Total number of synapses in the calyx
MBON-a1-R	4	305	309
MBON-a2-R	2	315	317
MBON-a1-L	0	222	222
MBON-a2-L	0	376	376

Table S2. Presynaptic and postsynaptic sites of MBON-a1/ MBON-a2 output regions around the MB medial lobe. 3D CATMAID reconstructions of MBON-a1-R, MBON-a2-R, MBON-a1-L and MBON-a2-L were used to count the number of presynaptic and postsynaptic sites in the ipsilateral and contralateral axonal branches in the output regions. Notice that the number of presynaptic sites is similar to the number of postsynaptic sites.

Neuron	Number of Presynaptic sites ipsilaterally	Number of Postsynaptic sites ipsilaterally	Number of Presynaptic sites contralaterally	Number of Postsynaptic sites contralaterally
MBON-a1-R	35	20	38	57
MBON-a2-R	33	36	38	63
MBON-a1-L	21	18	39	25
MBON-a2-L	46	65	35	49

Table S3. Reciprocal connections among_MBON-a1/ MBON-a2 in the output regions. These were counted using the number of synapses listed for each of the presynaptic partners of each neuron on CATMAID. For example, MBON-a2-R receives input from MBON-a1-R through three synapses, and MBON-a1-L receives input from MBON-a1-R through eight synapses.

		Presynaptic Neuron			
		MBON-a1-R	MBON-a2-R	MBON-a1-L	MBON-a2-L
Postsynaptic Neuron	MBON-a1-R	-	3	8	1
	MBON-a2-R	2	-	3	6
	MBON-a1-L	4	2	-	2
	MBON-a2-L	6	5	2	-
